# Ten simple rules for an effective mentor–mentee writing partnership

**DOI:** 10.1371/journal.pcbi.1014250

**Published:** 2026-05-06

**Authors:** Kristina Quynn, Megan J. Hemmerlein, Alexandra H. Keene-Snickers, Sarah M. Howard, Mark D. Stenglein, Kathryn Wilsterman, Carol J. Wilusz

**Affiliations:** 1 CSU Writes, Colorado State University, Fort Collins, Colorado, United States of America; 2 Cell & Molecular Biology Graduate Program, Colorado State University, Fort Collins, Colorado, United States of America; 3 Department of Biology, Colorado State University, Fort Collins, Colorado, United States of America; 4 Department of Microbiology, Immunology & Pathology, Colorado State University, Fort Collins, Colorado, United States of America; Dassault Systemes BIOVIA, UNITED STATES OF AMERICA

## Introduction

Shared writing projects are an integral part of graduate training in STEM disciplines; advisors work closely with their trainees on manuscripts, conference abstracts, proposals, and (eventually) a thesis or dissertation. Unfortunately, it is all too common for the writing relationship to be fraught with stressful interactions, which can lead to avoidance and inhibit learning for both the mentor and mentee. Indeed, one of the authors was spurred to work on their approach following the realization that their mentees were not growing sufficiently as writers during their graduate career to be able to draft their dissertations without significant oversight.

Our 10 simple rules are aimed at helping advisors and their trainees work more effectively together on writing projects, and are built on our experiences leading, evaluating, and participating in a collaborative writing workshop at Colorado State University. We also incorporated ideas and feedback from over thirty faculty and graduate students who have participated in this workshop to date. All the authors have also been engaged in mentor/mentee writing partnerships, some of us during the preparation of this article. While most of us are researchers with interests in computational biology, these 10 simple rules can be applied to many disciplines. Our goals are to describe the unique writing challenges that the advisor/advisee relationship creates, highlight pain points, and share rules that make the process of teaching and learning through writing more productive and enjoyable. We have also emphasized how Generative AI can be used, with caution, to support collaborative writing. To get the full benefit, we encourage you to discuss with your writing partner how you can apply these rules to your next project.

Any healthy writing collaboration begins with individual preparation and emerges through an open exchange of ideas, shared learning experiences, and intentional skill-building. Writing is a lifelong skill that researchers continue to develop over their careers, and even the most seasoned writers will find that co-writing forces them to confront their own shortcomings. This is in part because co-authoring involves a sophisticated set of disciplinary, rhetorical, and interpersonal practices: developing ideas, drafting, revising, negotiating, compromising, and much more. Co-authors often must work through collaborative writing processes many times over to produce a shared document that is ready for review and publication.

Collaborating on a writing project is difficult under most circumstances (addressed in another 10 Simple Rules article [1]), but the collaboration between a student and their advisor embodies additional challenges created by asymmetries in power, writing skills, and field expertise. Early-stage graduate students generally have minimal experience writing scientific documents, and their readiness to learn, confidence, and aptitude for the task will vary from student to student. Similarly, faculty experience with writing, mentoring, and mentoring *through* writing evolves with career progression and practice. Differences in cultural and linguistic backgrounds can present additional challenges, as can the fact that most scientific writing projects involve additional co-authors, not just a mentor/mentee pair. Each writing partnership is unique, requires time to develop and become functional, and must be adapted to fit each new project. Unfortunately, the challenges of writing together are not always recognized until after a bad experience, such as a rushed attempt to prepare a conference abstract due the same day, which may leave room only for frustration and hurt feelings. To set reasonable expectations and build trust, early attempts at writing together require careful planning as described in our rules below.

## Rule 1: Prepare to collaborate

Joshua Schimel reminds us in *Writing Science*: "As a scientist, you are a professional writer" [2]. Co-authorship is a great way to hone your writing skills. However, for mentor/mentee partnerships the inevitable differences in writing experience, disciplinary knowledge, confidence, cultural or linguistic background, and power make this a challenging relationship to navigate. A little preparation can go a long way in making the process easier.

As an advisor, you may benefit from reflecting on your writing experiences (see exercise in S1 File) to clearly define your guiding principles and philosophy. This exercise can support development of a lab handbook to articulate the lab norms on authorship and establish acceptable practices for writing in different genres (e.g., lab notebooks, protocols, code annotation, READMEs, etc.). A handbook should be a living document that is revisited and revised to reflect your growth as a writer and mentor.

Another often overlooked but invaluable way to share your perspectives is through reading and discussing the literature in your field. Many research groups meet regularly to review the latest advances, but it is less common to critique the writing in the articles. As an advisor, you can get writing value out of your existing meetings by encouraging your mentees to analyze how papers are structured, and to train them to identify and eventually emulate the characteristics of impactful, high-quality work. Computational biologists may want to take this further, by talking specifically about how code is documented and described [3]. The mutual understanding that comes from these conversations will lay a strong foundation to support implementation of the remaining rules.

**Fig 1:**
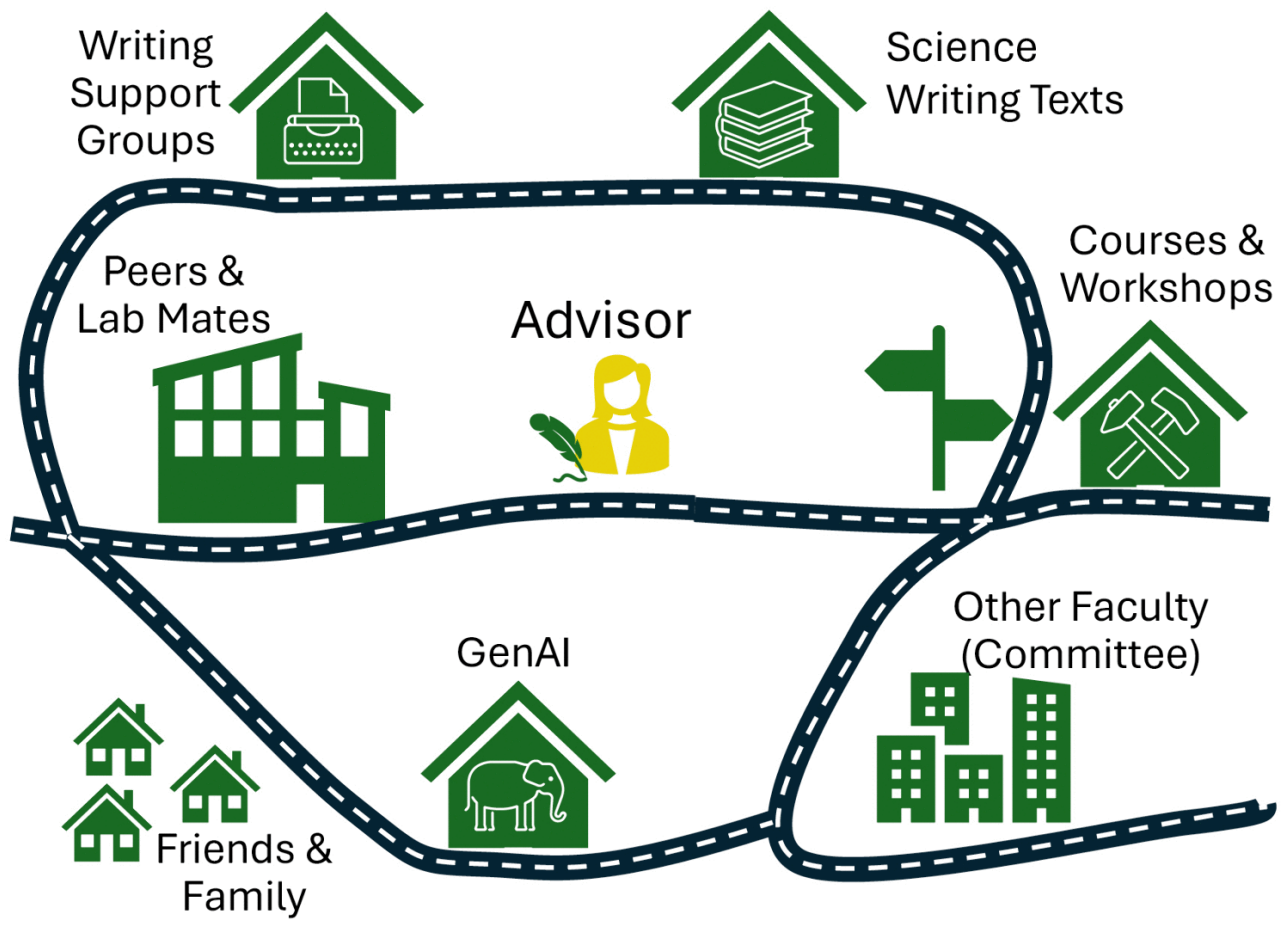
It takes a village to raise a science writer. There are many resources to provide instruction, support and feedback on writing to graduate students (and their advisors!). Leaning on these can reduce the burden on the advisor and allow the student to get a broader perspective on writing.

As noted above, co-authorship is a space to hone your skills rather than develop them afresh. As such, it relies on some basic competencies in writing and mentorship. During your first conversations, following completion of the reflection exercise (S1 File), you should identify training needs and corresponding resources to address them (Fig 1). Brushing up on your skills before you write together will save time and reduce frustration. Formal training can be immensely helpful, though the needs of mentors and mentees differ. Mentors may benefit from guidance in aligning expectations, communicating clearly, and facilitating the trainee’s writing development. The Center for the Improvement of Mentored Experience in Research (CIMER), for example, offers structured, evidence‑based modules that help advisors clarify roles, negotiate expectations, and build effective working relationships [4]. Mentees, on the other hand, often need to learn the fundamentals of scientific writing (see S1 File) or code documentation [3]. Many institutions and online platforms offer writing workshops, bootcamps, or courses in research communication, and several excellent science writing guides are available (S1 File).

Taking the time to get to know each other and build essential skills before you begin writing together will lead to a more confident and productive partnership.

## Rule 2: Set expectations early

Talking directly about roles, expectations, resources, and authorship can feel awkward—especially early in a writing partnership. Power dynamics can make it difficult for early-career researchers to raise concerns or express their preferences. Yet, having these conversations at the start of each new project will build trust and avoid conflict as you get to know each other.

Informal agreements can help with the day-to-day processes of writing together: how and when you will communicate, how you will share drafts and feedback, what tools you will use, and how long the process will realistically take. These informal agreements should also address goals and values associated with the project to make clear links between the writing process and their relationship to the project’s success. As collaborative writers, you can and should use these agreements to establish a productive rhythm and reduce friction during the writing process. It is natural for the advisor, as the more experienced partner, to take the lead here, but it is also important to give the student room to express what works best for them. Both of you will find spelling out the expectations and revisiting them along the way smooths the collaborative process. Keep in mind that neurodivergent writers and English language learners may carry a heavier cognitive load and approach writing, feedback, and deadlines differently. (See *Collaborative Writing Guide* (S1 File) for more information).

Informal writing agreements are generally not a one-and-done activity. Over time, you will need to reflect on how your partnership has matured, gauge improvement, and recalibrate your expectations. Returning to these discussions will almost certainly result in a more efficient and effective writing process.

For long-term, or high-stakes projects and those with many collaborators, formal coauthor agreements can provide structure and clarity. These agreements should address areas such as author contributions and order, AI use (see Rule 3), and data management and sharing [5]. You can build your own template based on journal/funding agency/institution guidelines or use an existing template such as those we provide in our guide (S1 File). While you may feel coauthor agreements are overly formal, they serve as shared reference points, help prevent misunderstandings, and keep all parties’ best interests visible.

## Rule 3: Spell out the role of generative AI

**Fig 2:**
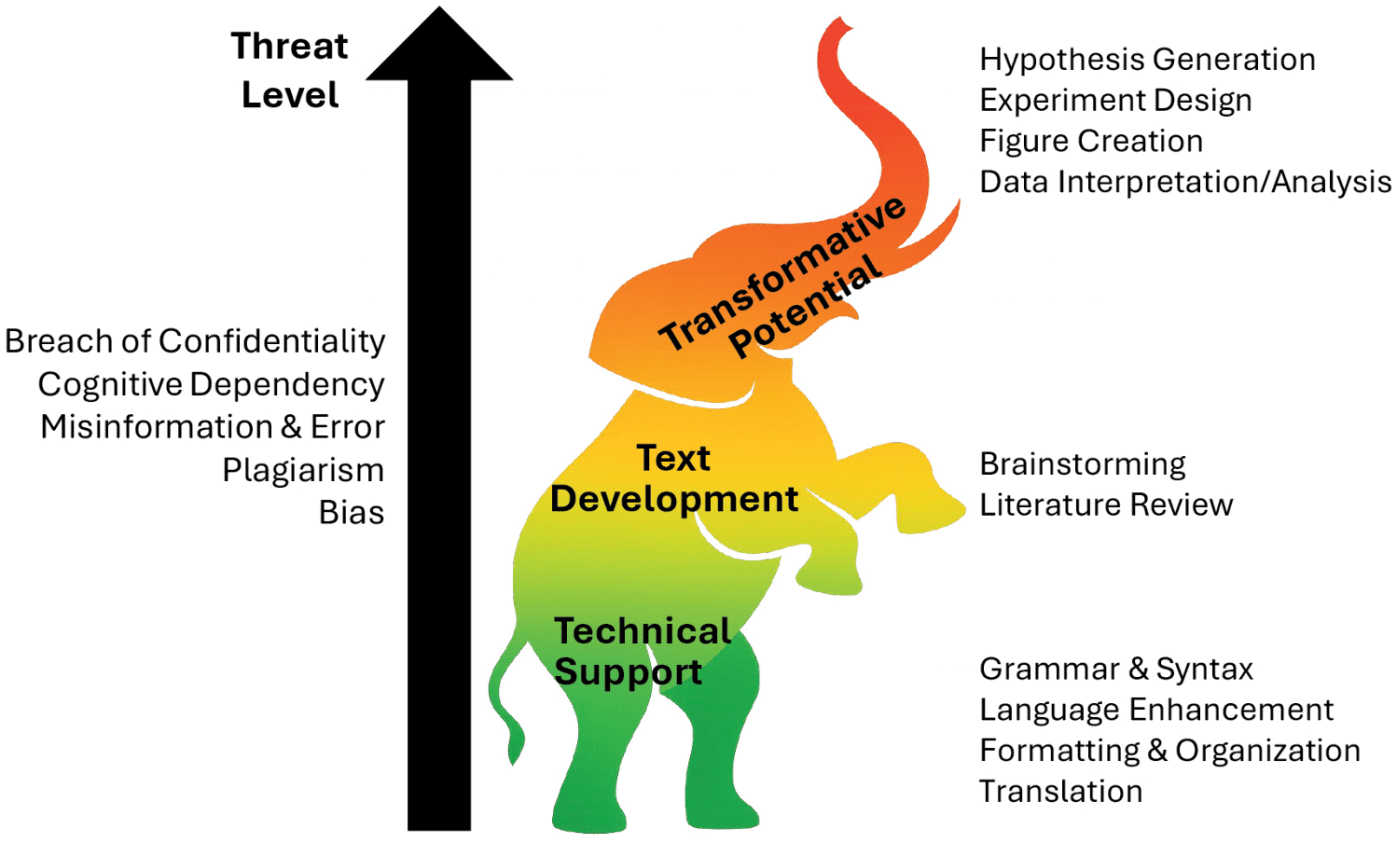
Ignore the GenAI elephant in the room at your own risk. Low risk applications such as correcting grammar and syntax can save a great deal of time, are embedded in your word processor, and if used appropriately can also enhance learning. Using GenAI to perform higher level tasks is potentially transformative for those who have already mastered their field, but it can allow trainees to bypass learning that is key to their future scientific success. You need to balance the benefits with the drawbacks and find your own comfort level. Figure based on the 4Ts pyramid [6]. Elephant was generated using MS CoPilot.

Due to the associated risks and transformative potential (Fig 2; [6]), we feel GenAI warrants its own rule although it should certainly be addressed as part of your discussions associated with Rule 2. You may both have more questions than answers about how to use Large Language Models (LLMs) effectively and ethically, and it is very possible that the student may have more GenAI experience than the advisor. This inversion of experience is not surprising, as researchers at all career stages are acquiring AI literacy on the job with minimal training and few official guidelines. It is therefore imperative to specifically address AI use (and not treat it as the elephant in the room (Fig 2)). The mentor should still be guiding and evaluating the trainee to ensure that GenAI is being used to support or enhance learning rather than bypassing it altogether. To avoid misunderstandings and undesirable outcomes we strongly recommend you implement a living “AI-Use Agreement” to clearly define what tools may be used, at which stages of document production and for what purposes. We are providing a template (S2 File), and some resources (S1 File) here, but you will both need to invest some time to first explore and then discuss the capabilities and limitations of LLMs and other platforms you plan to use to support your writing. You should also make sure your use of AI aligns with any guidelines provided by the intended publisher or funding agency. Articles providing classifications and core principles may be a useful starting point to guide your discussions [7,8] and recent Ten Simple Rules articles cover AI use in research and writing in more detail [9–11]. You may also want to touch on the social, economic, and environmental costs of using this technology [12]. Our take home message is: if you jointly decide to use GenAI for your writing, it is vital that you (1) agree upon, and are transparent about, which platforms can be employed and how, (2) recognize you both remain 100% accountable for the output, including errors, misinformation, bias and hallucinations (a final “human review” is essential!), and (3) take precautions to ensure your unpublished data, intellectual property and innovative ideas remain secure.

## Rule 4: Start small to build confidence

Writing can be time-consuming and anxiety-inducing even in the best circumstances. For trainees, particularly those early in their writing career, the idea of handing in any type of writing to their mentor can be paralyzing [13]. One way to ease this stress is to begin early with low stakes or short assignments to reduce the pressure to produce perfect text [14]. This strategy allows the mentee to iteratively improve their writing skills and learn to receive feedback without taking it personally. Low-stakes assignments should require relatively little time and can take a variety of forms, including paper outlines, READMEs, conference abstracts, manuscript sections that are straightforward to draft (e.g., methods, figure legends), or content not destined for publication (coursework, protocols, lab notebooks, research summaries, reports). As the mentor–mentee writing relationship progresses, the complexity of writing assignments should also progress. Perhaps as data are generated and a story begins to unfold, the mentee can generate text to go along with figures and share these for feedback. This approach not only gradually introduces higher stakes writing but also ensures that the mentee does not spend hours on text that does not meet requirements. Every assignment represents an opportunity to practice and grow, providing space to discuss the processes and practices of writing as well as supporting development of a working relationship where you each understand your partner’s expectations.

Adopting an iterative draft-feedback process can also reduce the stakes of writing (Rule 8). Mentors should emphasize that first drafts are just that: the first of many drafts that will improve over time. The prospect of turning in a draft of some text is less daunting than turning in a presumably complete version. Mentees should not lose sight of the value of a draft, even a rough one.

## Rule 5: Own your part of the project

Owning your part of a writing project requires that you first understand your responsibilities (Rule 2), which will be distinct for each advisor/advisee duo. The next step is determining what best practices you can apply to solidify ownership of your contribution.

Students will increasingly recognize and learn to edit errors in their writing. Self-editing is a skill developed through practice—ideally with low-stakes assignments like those mentioned in Rule 4. The key to successful self-editing is timing. Especially in early drafts, students may default to comfortable patterns: familiar styles, passive voice, run-on sentences, all the while ignoring the red underlines from the word processor. For generating initial rough drafts, ignoring autocorrect is a completely acceptable and even encouraged method for getting the creative juices flowing. If you self-edit while getting first words onto the page or while organizing your thoughts, you might never make any progress. However, submitting a draft containing overt errors to your mentor for review can hinder the feedback process. Consider the frustrating experience of the advisor who repeatedly highlights similar sentence-level errors or the same conceptual concerns across multiple drafts. Self-editing should be applied after logically organized paragraphs are on the page, but before you send the draft to your writing mentor. Build time into your schedule to step away from your draft (at least a few hours, or even a few days) and revisit it with a critical eye. As the mentee, put on your mentor hat and ask yourself, what will my mentor focus on? The wording of my hypothesis? The difference between “stained” and “labeled”? My four-line-long sentences? The fact that I am still using “Because” to start a sentence even though I know they hate it? (a pet-peeve of one of the authors). Self-editing should be rooted in the conversations about quality research writing that you have had with your mentor and explored by reading model articles.

Your goal is to identify problems as they crop up in your writing and eventually avoid making them in the first place. Thoughtful and thorough feedback requires time and energy, and if the lead writer is not tracking and learning from prior feedback, focus will invariably be drawn away from other elements of the draft that need attention. Grammar and spelling improvements can be supported through functions that are now built-in to most word processing software. A skills checklist or an error-tracker can highlight recurring sentence-level and word choice issues. (S1 File). If all coauthors have agreed, you may consider using GenAI and/or other resources (Fig 1) to direct your self-editing process prior to sharing a draft with your advisor. However, keep in mind that using GenAI can place your data, copyright, or learning at risk (see Rule 3).

As the advisor, owning your part of the project involves setting a good example for best collaborative writing practices, which includes sticking to agreed-upon deadlines, communicating proactively (Rule 6), and giving your partner time to implement changes or make progress (Rule 7). Actively engaging in mutual respect and communication is essential to maintaining trust around shared goals, especially when the writing process gets bumpy.

**Fig 3:**
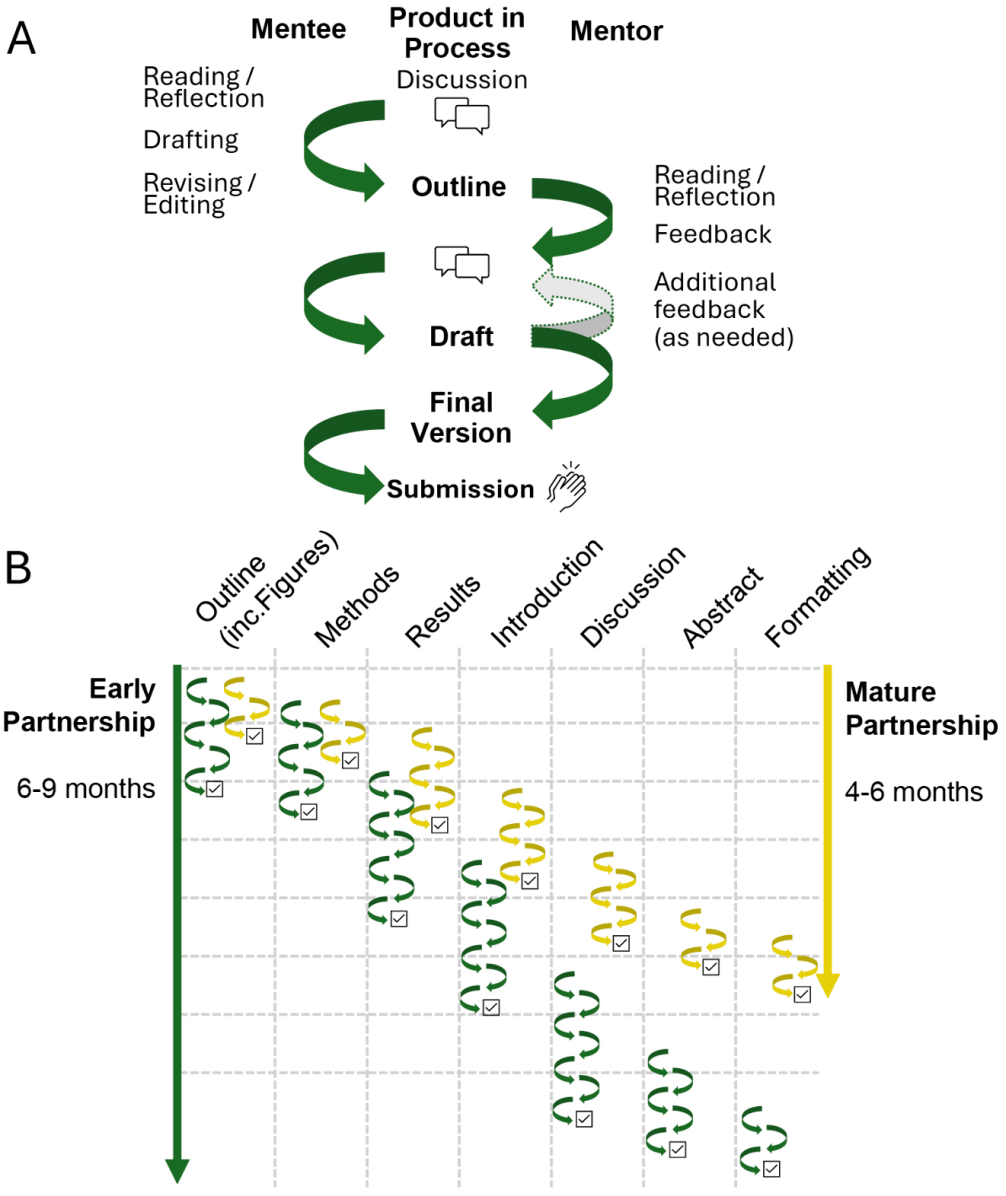
Process and timeline for co-producing a scientific manuscript. Panel A depicts the collaborative process for a single section of a manuscript or a small-scale project such as a conference abstract. The cycles of drafting, reviewing and revising all take time and effort for both partners and multiple exchanges are required to get to a final document. For an abstract, if both parties are focused it may take 2-3 days to get to a final product, but a larger project like a thesis chapter could take a month or more. Panel B shows how this process is repeated to complete a manuscript. Progress can be expedited if each of you work on different sections at the same time (e.g., the mentee starts on the Methods section while the mentor reviews and comments on the Outline), but this requires clear communication about the status of each section and feedback needed. Panel B also highlights how the process becomes more efficient as you become more adept at working together (compare green, early partnership, to gold, mature partnership).

Mentors will also find it helpful to extend the goal from finishing one project to providing an environment for your mentee to improve as a writer and as a thinker (i.e., intellectually). Taking the time to coach the mentee in their writing promotes confidence that extends well beyond submission of a single manuscript or proposal (Fig 3).

## Rule 6: Communicate clearly when exchanging drafts

The process of exchanging drafts and giving/receiving feedback is inherently long (Fig 3), but collaborative writers can streamline this by starting on the same page about the stage of the draft and the type of feedback it needs. Initial versions—the proverbial “crappy first drafts”— usually benefit from more *interactive* comments and questions to encourage the framing of ideas and improve organization. At later stages, *corrective* feedback will fine-tune language and make the draft clearer or more concise. Understanding the different types of feedback (Box 1 [15]) and how they can be helpful at different stages of idea and manuscript development can help you work together more effectively.

The different sections of a manuscript will inevitably progress at varying rates—the methods section may be completed before anyone even starts the introduction; often, we (as writers) revisit the results to add new analyses as part of the process of honing the larger story (Fig 3). This natural asynchrony means that multiple sections of a writing project may be ready for different levels/types of feedback at the same time. The mentee should proactively communicate clearly the type of feedback needed every time a document is submitted for mentor review. Otherwise, you may end up receiving detailed corrective edits where you were hoping for guidance on big-picture concepts, or you may receive limited feedback on the discussion simply because it languishes at the end of the document and is read only superficially.

**Box 1. Types and stages of feedback based on feedback definitions from Purdue OWL’s *Faculty Guide for Working with Graduate Writers* [15].**

**Table:** 

Type of feedback	Definition	Recommended stage
Interactive	Partners have a conversation about the draft (either in person or “in the margins”). The mentor asks questions, highlights areas of confusion, explains problems, and makes suggestions. The mentee is left with much to do, including reflecting on the concerns, asking for clarifications, deciding how to address the issues and finally revising.	Initial stages of any project to develop and arrange ideas; Initial and mid-stages when the student has less experience, to foster learning.
Directive	The mentor points out specific problems and may offer detailed suggestions for correcting, but does not make the corrections; the student must apply the suggestions.	Initial and mid-stages as the student gains confidence and becomes better at addressing comments.
Corrective	The mentor makes corrections on the page; the student accepts or rejects the changes.	Later stages to polish ideas and to increase accuracy, precision, and readability.
Evaluative	The mentor makes a judgement call and indicates that something in the text is well written or needs attention (e.g., *I like this*); the student may learn from, address, or ignore such comments.	All stages to highlight the quality of writing and ideas. Use with care because making “judgements” can communicate an emotional response rather than practical advice for revision or further consideration.

One effective tool for focusing your partner on the stage of the draft and feedback needed/given at each exchange is a cover letter-style email or message accompanying the draft. A cover letter need not be formal (though it can be good practice) and at its most succinct simply describes: (1) the contents and status of the draft, (2) what is strong or working well, and (3) what requires work or guidance.

The cover letter allows mentees to communicate where they have put focused significant effort, to assess the status of manuscript sections (e.g., idea organization, fleshing out, or ready for copy-editing), and to clarify the type of feedback they are looking for. This structure encourages reflection and clear communication (instead of just pushing the manuscript into someone else’s inbox – we have all been there).

Mentors can use their cover letter to direct editorial effort by focusing on what the mentee needs to know to move the manuscript to the next stage. Note that it can be difficult to limit your edits to those requested, especially if you notice significant problems in other areas of the draft, or if your writing process differs from your partner’s. So, try to remember that being responsive to the requests of your partner builds resilience and trust in your relationship by acknowledging each other’s process and your common goals. Staying focused also minimizes the chance of feedback exchanges that feel too personal or too extensive for a student to engage with [see Rule 8 for more on this]. You may also find a feedback cover letter helpful to summarize and explain your editing process when returning the document (See template in the *Collaborative Writing Guide* S1 File).

Ultimately, cover letters provide both mentor and mentee an opportunity to provide an assessment of the project’s status with each exchange of the manuscript (e.g., idea development, fleshing out, organizational edits, proofing, or copy-editing stage). Places where you disagree on the stage of development are important to discuss, both for the growth of your writing relationship and to avoid future frustration. Assessing manuscript stages also provides space for the mentor to gently point out areas that may be a concern in the future rather than overwhelming the mentee with edits that they may not yet be ready to receive or implement.

## Rule 7: Allow time for multiple rounds of feedback

Even highly skilled writers who have been practicing for decades spend significant time reorganizing, rewriting, and editing their work. Ernest Hemingway wrote over 40 endings for his 1929 novel, *Farewell to Arms* [16]. The number and extent of revisions may come as a surprise to those just starting their writing career and is therefore worth stressing early on. The first draft is never the final draft, nor, for that matter, are the second or third. All of this takes longer if you are working with a collaborator or team, and perhaps longer still if the lead writer is an early-career developing writer (Fig 3).

As a mentor, you might be tempted to make extensive changes to get a document “out the door” but wholesale re-writing without adequate context limits the opportunity for learning. Ensuring that there is enough time for you to formulate and explain feedback and for the student to digest and respond is key to the learning process.

How long is *long enough*? The answer depends on the scope of the project, but it is safe to say that the process of crafting a submissible quality manuscript will take longer than you think. Humans consistently underestimate how long it will take to complete a project. One landmark study compared how long it took students to write their theses to how long they had expected it to take [17]. Writing time surpassed even their worst-case projections, which had assumed that “everything went as poorly as it possibly could.”

A suggested timeline for writing a scientific paper is presented in Fig 3. This workflow includes several rounds of feedback per document subsection, with each round containing time for mentee writing/editing and mentor feedback (Fig 3A). We [AHKS, MDS] have found that one week is sufficient for the mentor to provide constructive feedback focused on guiding the mentee (rather than rewriting which might be quicker). Mentors can make a standing commitment to return drafts within a specified time (for example, within one week). This clear expectation helps mentees plan their own schedules and keeps projects moving forward. You can adjust the timeline and order to fit the complexity of the project, your personal preferences, the maturity of your partnership, and any approaching deadlines. You also need to consider predictable confounders like travel, teaching, classes, medical appointments, and childcare. Being transparent about other commitments and adjusting the timeline to accommodate them goes a long way in reducing the stress associated with writing together.

The timeline presented focuses solely on a mentor/mentee partnership working to produce a manuscript. In most STEM fields, two-author publications are relatively uncommon, so bear in mind that the process becomes more complex, and the timeline will extend when additional authors are involved, especially if these are external collaborators. We refer readers to another Ten Simple Rules article for guidance on how to navigate these larger writing projects [1] with the additional note that trainees will need mentor support and advocacy to contribute effectively as part of a larger group.

## Rule 8: Provide and receive feedback thoughtfully

As a faculty member, you will have developed your own voice and strategies for writing which must be working at some level, given your success so far. However, much of your acumen may have been acquired through implicit learning, making it hard for you to describe how and why you write the way you do [18,19]. It might require considerable effort to provide constructive feedback and to explain recommended and necessary edits, but trainee science writers will learn much more through the process than by having their mentor “fix” the problem (corrective feedback—see Rule 6).

As a graduate student on the receiving end of your advisor’s “wisdom,” you may find it disheartening to receive a heavily marked-up document bearing little resemblance to the one you submitted, especially if you had invested a lot of effort to create what you thought was a solid draft. In such moments, you might be tempted to take an easy out by clicking “accept all changes” rather than putting in the effort needed to understand the reasons behind the edits. Avoid this impulse and refer to Rule 4 to track feedback and accelerate your writing skill development.

Heavily edited manuscripts and “accept all changes” are common, and while clearly not ideal, can lead to a finished project (one measure of success). However, the chance of learning can be lost. You risk your partnership being trapped in an unproductive cycle that is frustrating on both sides and doomed to repeat itself with subsequent projects.

Box 2 provides some strategies to smooth the path of giving and receiving feedback. These strategies are not one-size-fits-all. Both of you will need to adapt and compromise to make the process work. As a mentor, you will find some students respond well to direct and unequivocal statements, while others need to have feedback foregrounded by additional context. You may need some time to figure out what works best, and this may change as your trainee matures. Student writers should start with the understanding that as collaborators you want the same things—a well-written document and supportive research relationship. This assurance will help you receive and respond to feedback objectively. So, too, will meeting in person to go through edits together (interactive feedback) to clarify areas of confusion or disagreement. Concentrate on understanding, responding effectively to, and learning from suggestions, and the writing process will eventually require less effort. Time invested in developing mutual trust and learning each other’s styles and preferences during the early stages of your partnership will pay dividends when you embark on writing your first paper together.

**Box 2. Tips for giving and receiving feedback effectively.**

**Table:** 

Giving feedback (mentor)	Receiving feedback (mentee)
**Use a respectful and supportive tone** It is easier to hear polite suggestions, and it costs little to modify your language.	**Take a deep breath** Stay open-minded and positive. Adopt a growth mindset. Criticism is not personal - it is intended to help you build skills and improve the final document.
**Be specific** Avoid cryptic comments. Explain why you are making a change or suggestion. If you can’t explain, then maybe the change is subjective and not necessary.	**Notice strengths** Be kind to yourself. Even an absence of edits can be taken as a positive island in a sea of corrections!
**Avoid just fixing the problem—suggest solutions** Pointing the trainee in the right direction gives them agency in addressing the concern.	**Pay attention and reflect before responding** Don’t make changes without understanding and agreeing with them. No “Accept All Changes”. If you are feeling frustrated, then take a break before responding.
**Balance criticism with praise** Highlighting evidence of mastery or improvement helps the trainee stay motivated.	**Ask clarifying questions** This can help your partner understand the kind of feedback that you need and reduce the number of rounds of editing.
**Ask questions** Guide the student to the correct response.	**Express gratitude** Even negative feedback takes time and effort.
**Allow time to be thoughtful** A rushed critique will rarely be balanced or complete.	**Learn from the advice** Don’t waste your advisor’s time by making the same mistakes again. Keep track of issues and patterns in your writing, both good and bad.
**Minimize subjective changes** Distinguish between edits that are correcting errors versus altering the style to match your own.	**Stand up for your voice** You are an author too. If the edits are a matter of style, then sometimes it is ok to stand your ground.

## Rule 9: Be solution-oriented and willing to compromise

A productive writing relationship ultimately depends on both mentor and mentee being invested in finding a path forward for the project. You will inevitably disagree on many things. Some differences of opinion may impact the final product significantly, such as whether data is too preliminary, if the importance of a result is over-stated, or whether the findings in a pre-print should be cited or ignored. Other disagreements, such as whether the writing style is too conversational, or the background information too extensive, may have lower stakes depending on the journal and audience. Disagreements over minutiae like font sizes in figures and naming conventions can be easily resolved by referring to the journal and/or field policies. Due to the inherent power dynamics at play in your relationship, mentees may feel pressure to acquiesce to the mentor’s perspective. Indeed, in many cases, the mentor’s suggestions are grounded in experience, and thus their apparent inflexibility or pickiness can be well-justified. However, writing together is a critical opportunity for the mentee to practice and develop academic insights and independence, so an overreliance on the mentor’s perspective or direction can inhibit learning. As such, both of you must be proactive in seeking joint decisions to resolve differences of opinion.

Being solution-oriented requires both action and attitude. As described in Rule 8, articulating the rationale for your decision-making allows you to focus on specific points of contention and develop solutions from a shared starting point. For mentors, solution-oriented action comes through the types of feedback you provide (see Rule 8). Rather than only pointing out problems, consider explaining your position and providing (multiple) potential remedies. This approach has many benefits: it promotes ownership and independence, it reminds you both that there is more than one way to effectively communicate your ideas, and it encourages talking through the pros and cons of each solution to get to the best one. For mentees, putting forward multiple solutions will be a powerful tool for directing your own learning and eliciting your advisor’s input. Mentors must be willing to separate professional identity from the writing and recognize that the goal should not be to create an intellectual “mini-me”. One of the strongest signs of a healthy writing partnership is evidenced by a mentee having confidence to bring ideas to the table that enrich the writing or build new connections [20]. If mentors do not promote exploration, mentees may struggle to develop the independence of thought, writing skills, and scientific voice that they need to be successful in their next career stage.

If your views are not aligned, seek out independent opinions (e.g., from other lab members or a trusted collaborator), reference the journal’s guidelines, or consider moving controversial information to the supplementary section. It is also okay, and sometimes beneficial, for a mentee to learn through peer review or to suffer the heavy editing from another co-author at a late stage of manuscript development. Who knows? Solution-oriented compromise can also serve mentors’ ongoing development when they realize a stubbornly held opinion was ultimately not of major importance to others.

## Rule 10: Practice patience and compassion

Approaching collaborative writing projects with patience and compassion for each other is essential. This rule is perhaps the most important because it influences how effective you will be at implementing Rules 1 through 9. Writing together should not be a task on a to-do list, but rather a mutually rewarding opportunity to grow and learn. The emphasis on growth and learning is a key distinction between mentor-mentee writing and purely productivity-driven writing. Recognizing this, and that your partner is working in the face of competing demands and pressures to be productive, will strengthen your collaboration.

As a mentor, you should remember that training a good writer takes time, often more time than you anticipate or want to allot. Indeed, the effort devoted to writing is a key component of mentoring. Ideally, the time invested will pay off as your student grows into an increasingly independent and capable writer. You can practice compassion by understanding that writing is stressful, and it may take time for your student to feel comfortable sharing their work, receiving feedback, and working through the editing process. Setting expectations and agreeing on a timeline at the early stages is critical (Rules 2, 3, and 8). To that end, you would do well to reflect on where you might need to improve your mentorship and writing skills and seek out resources (Rule 1).

As a mentee, practice patience with yourself because becoming a good writer takes time and a lot of effort. You might find it overwhelming to receive and respond to numerous edits, comments, and suggestions. Take breaks and adopt a growth mindset, reminding yourself that processing and responding to feedback is a crucial component of your professional development [21]. Remember that feedback is not a reflection of your intelligence or character but rather a manifestation of your mentor’s faith in your potential. Finally, you should also bear in mind that even for an experienced advisor, providing thoughtful feedback on a document takes time and their time is usually in high demand. Planning and patience are key.

## Conclusion

We hope these 10 Rules have provided some useful resources and perspectives to smooth the path of your next shared writing project.

These rules are relatively easy to implement if both partners are motivated to prepare in advance, communicate clearly, and show compassion. While following our rules may require you to slow down your initial attempts at co-authoring, you will regain this time and much more in the later stages of training. There will be many ancillary benefits: a stronger mentoring relationship, better quality (and more) manuscripts and proposals, and less stress associated with the writing process. We wish you both a productive journey through engaging writing conversations to manuscripts and proposals you can be proud of.

## Supporting information

S1 File Collaborative writing guide. This guide contains additional guidance and resources for faculty and students engaged in collaborative writing.
(PDF)

S2 File Co-author AI agreement. A template for developing an agreement on allowed uses of AI during preparation of a manuscript or proposal.
(PDF)
