# Supplementary material for: Ten simple rules for an effective mentor–mentee writing partnership
Source: PLoS Comput Biol. 2026 May 6;22(5):e1014250. doi: 10.1371/journal.pcbi.1014250 (PMC13148713; doi:10.1371/journal.pcbi.1014250)
Supplement: S1 File — This guide contains additional guidance and resources for faculty and students engaged in collaborative writing. (PDF) [file pcbi.1014250.s001.pdf]

Collaborative Writing Guide

Advice, Exercises, and Resources for Mentors and Mentees

CSU WRITES
COLORADO STATE UNIVERSITY

CELL AND
MOLECULAR BIOLOGY
COLORADO STATE UNIVERSITY

Table of Contents

Acknowledgements 4

Reflecting on Research Writing Experiences 5-6

Understanding Collaborative Writing 7

Skills Checklist for Competent Scientific Writing 8

Advice for Building Competency in Scientific Writing 9-10

Fostering a Writing Culture 11

Writing with Multilingual and Neurodivergent Coauthors...............12

Developing Collaborative Writing Agreements 14-15

Coauthor Agreements 16

Team Agreements 17

Understanding Types of Feedback 18

Requesting Feedback.......................................……..................... 19

Communicating about Feedback…………………………….…. 20

Writing Logs 21

Error Trackers 22-23

Resources 24-26

©2025 CSU Writes and Cell & Molecular Biology, Colorado State University
All Rights Reserved
Colorado State University grants permission to all educational institutions to copy and use any material contained in this guide with proper citation.
Content compiled and developed by Kristina Quynn, Ph.D.
For more information, contact CSU Writes at: csuwrites@colostate.edu or go to: CSUWrites.ColoState.edu

ACKNOWLEDGEMENTS

We are grateful to the faculty, postdoctoral scholars, and graduate students at Colorado State University who participated in the *Mentoring through Writing* workshops from 2020 to 2023. This handbook and the accompanying workshop series were developed and have been continually refined based on your thoughtful feedback, insightful questions, and evolving needs. You have been engaged participants, generous scholars, and inspiring collaborative writers. Thank you.

Mentoring through Writing was a collaborative effort of the directors of CSU Writes and the Cell and Molecular Biology program, whose shared commitment to fostering a culture of productive research writing and scientific mentorship made these workshops possible.

We especially recognize the value of interdisciplinary collaboration that emerged through this partnership. The diverse perspectives and disciplinary approaches of participants enriched our workshop conversations and the collaborative mentoring through writing practices that inform this handbook.

This Collaborative Writing guide and workshop (2020-2023) were supported by funding from a CSU Graduate School Center of Improvement of Mentored Experiences in Research (CIMER) grant and a National Institutes of Health T32 training grant (GM132057).

Kristina Quynn, Director of CSU Writes
Carol Wilusz, Director of Cell and Molecular Biology

FACULTY & PI MENTORS

EXERCISE: Reflecting on Research Writing Experiences

Research writing is highly constructed and its production, demanding. As experts or becoming-experts in a field of study, we gradually hone our skills through a variety of writing experiences with mentors, instructors, editors, and colleagues.

Reflecting on this professionalizing process can enhance our metacognitive awareness by illuminating the often-hidden processes, assumptions, and expectations we bring to writing. The better we understand ourselves as field experts and professional writers, the better equipped we are to support the scholarly development of graduate students.

Consider your responses to the collaborative writing topics and questions below. When you meet with your mentee to discuss your writing philosophy and mentoring background, select some of your reflections to share so that they can better understand and connect with you as a field expert, writer, mentor, and collaborator.

1. **Describe your approach for working with graduate students on their writing.** How formalized or structured is your process? How explicit are your instructions? How often do request drafts? How often and in what manner do you provide feedback?

2. **Recall a valuable writing training experience with a mentee.** What part did you play in the crafting of the document? What made the collaboration successful or meaningful?

3. **Reflect on a time when a writing mentorship experience did not go well.** What were the circumstances and challenges? What did you learn from the experience?

4. **What do you understand to be the consequences of not having enough time to improve the quality of scientific writing?**

5. **What are outcomes (positive and negative) of “fixing” graduate student writing?** What are the potential benefits? What might be lost in the process?

6. **How have you used generative AI or other software (e.g., ChatGPT, Grammarly, DeepL, Googledocs, Dedoose, or others) in your own research writing and mentoring practices?** What are your thoughts on the benefits and limitations of writing and data analysis tools in the context of research writing and graduate training?

GRAD & DOC MENTEES

EXERCISE: Reflecting on Research Writing Experiences

Research writing is highly constructed, and its production is demanding. As experts or becoming-experts in a field of study, we gradually build and continually hone our writing skills through a variety of experiences with mentors, instructors, editors, and colleagues.

Reflecting on this professionalizing process can enhance our metacognitive awareness by illuminating the often-hidden processes, assumptions, and expectations we bring to writing. The better you understand yourself as a developing field expert and professional science writer, the more effectively you will communicate with your PI or faculty mentors.

Consider your responses to the collaborative writing topics and questions below. When you meet with your PI or mentor to discuss your research writing background, select and share relevant reflections to help your mentor better understand and work with you as a learner, writer, and collaborator.

1. **Describe the process of producing a manuscript in collaboration with previous (or your current) faculty mentors and advisors.** How formalized or directed was the process? What did you find most helpful in those learning or collaborative writing experiences?

2. **What are your strengths as a writer?** Consider: What have other said they like about your writing? What in your writing practice has served you well to date? What do you enjoy most about writing?

3. **What are some areas you would like to improve?** Consider: Are there patterns in the feedback you have received in the past? What in your writing practice does not serve you well? What do you like least about writing? Is there something here you wish to address?

4. **Describe an instance of writing feedback and support that helped you grow as an academic or research writer.** What was the project? What kind of feedback did you receive? What about the process was most helpful?

5. **What do you understand to be the consequences of not having enough time to improve the quality of scientific writing?**

6. **What are outcomes (positive and negative) of your PI or faculty mentor "fixing your writing?** What are some of the potential benefits? What might be lost in the process?

7. **Do you use generative AI or other software (e.g., ChatGPT, Grammarly, DeepL, Googledocs, Dedoose, or others) to support your writing and analyses?** If so, how have these tools helped or hindered your research writing process? What are your thoughts on their role in your development as a scholarly writer?

Understanding Collaborative Writing

Collaborative writing in scholarly and scientific contexts takes many forms. Most commonly, it refers to the creation of a **single text by multiple authors**, but it can also describe situations where writers work **independently on separate texts** within a shared writing environment.

This section outlines key types of collaborative writing, including synchronous and asynchronous methods, as well as different team-based approaches to drafting and revising. Understanding these distinctions can help you navigate collaborative projects more effectively when you are coauthoring a manuscript, contributing to a grant proposal, or working within a research group.

Synchronous Writing
refers to the processes by which two or more writers work on the same document, section, or sentence at the same time. Writers may be engaged in drafting, editing, adding data and figures, tinkering with a bibliography, or any other tasks typical of building a manuscript. The document phase does not define *synchronous writing*, rather the definition hinges on writers working on document at the same time.

Asynchronous Writing
refers to the process by which two or more writers work on the same document at different times. Writers will pass a document back and forth (for pairs) or sequentially (for a team) to build, revise, and edit. Historically, the document would be generated and passed in hardcopy. Today, we commonly add to digital documents, use track changes and comments in the margins, and forward by email to our collaborator(s). Asynchronous is common to collaborative writing among academics, faculty mentors and their student mentees.

Collegial (or Lead Author) Method
refers to a mode of collaborative writing in which one research writer takes the lead on generating and compiling a working document for the group. The partner or rest of the team will provide editorial and field expertise in the shaping of the final document.

Sequential Method
refers to an asynchronous process of document drafting and revising in which each writer contributes their section(s) before forwarding to the next to add or edit (Lowry, et al. 2004).

Parallel Method
refers to the arrangements of synchronous or asynchronous processes for document generation in which writers produce designated sections of a document. When writing synchronously, collaborators may gather in a room (physical or virtual) to speak and write sections while one-member (lead author, scribe, or a subgroup acting as lead) compiles (Ede and Lunsford 1990, Lowry et al. 2004).

Reactive Method
refers to an arrangement of synchronous generative and revision processes through which writers create a document in “real time” (Lowry et. al 2004).

Mixed Mode (for Collaborative Writing)
refers to the strategic use of more than one of the collegial (lead author), sequential, parallel and reactive methods during the phases of drafting and revising their document.

Lowry et al. 2004. “Building a Taxonomy and Nomenclature of Collaborative Writing to Improve Interdisciplinary Research and Practice.” https://doi.org/10.1177/0021943603259363.

GRAD & DOC MENTEES
Skills Checklist for Competent Scientific Writing

Use the list below to identify the writing skill competencies about which you feel proficient or **CONFIDENT (C)** and those competencies you are **DEVELOPING (D).**

After self-assessing, select 1-2 developing competencies that would most improve the quality of your writing. Focus your time/effort on those to see immediate speedy progress.

C ☐ **D** ☐ **Understanding of Expected Document Organization and Structures**
Most fields have accepted formats for documents like abstracts, manuscripts, reports, grant proposals. Take note of the preferred formats before you start writing!

C ☐ **D** ☐ **Ability to Craft Writing that is Both Clear and Concise**
Scientific writing needs to convey complex concepts. Extraneous words and phrases that do not convey useful information should be avoided. Complex words should be avoided if a simpler or more familiar word can convey the same meaning. Sentences should be short and simple. Avoid redundancy and irrelevant details.

C ☐ **D** ☐ **Mastery of Field Specific Vocabulary**
Each field has its own specialized terms/jargon. It is important to use these words appropriately when writing for the expert (but to avoid or define them when writing for a lay audience). Remember to define acronyms.

C ☐ **D** ☐ **Knowing When to Use Passive versus Active Voice**
In general, the active voice is preferred (e.g., "We wish to suggest a structure for the salt of deoxyribose nucleic acid."). Methods sections, however, more commonly use the passive voice (e.g., "A structure is suggested for the salt of deoxyribose nucleic acid.").

C ☐ **D** ☐ **Crafting Sentences with Precision and Accuracy**
Words like "significance" and "correlation" have precise statistical connotations and must be used with care. Be quantitative – "mRNA abundance increased 20-fold" is more informative than "mRNA abundance increased." (Note: Currently, GenAI summaries often provide misleading or blatantly incorrect information and summaries of scientific and scholarly publications—often because their predictive generation of information presents what is plausible, not what is accurate. Take care when using AI summaries of literature and in reviews of your writing.)

C ☐ **D** ☐ **Writing to a Scientific Audience Means Minimizing Figurative Language**
Use metaphors and similes with care. Avoid clichés, puns, and hyperbole.

C ☐ **D** ☐ **Synthesizing Information**
Avoid listing facts from different sources. Summarize and make connections for the reader. (This skill is the essential work of research and scholarly writers.)

C ☐ **D** ☐ **Including necessary Transitions**
Make logical connections between ideas and paragraphs.

C ☐ **D** ☐ **Correct use of Sources and Citations**
Know where and how to cite your sources. In general, you should be citing primary research articles. Review articles should only be cited when the topic is tangential to the central theme. Try to avoid listing more than two or three citations to support a single statement. Use a reference manager (Zotero or EndNote).

Basic Skills Reminder for All Writers

Foster a craft-to-expertise approach to continually develop skills across your career span. Excellent science writers and communicators continue to grow from early through advance career stages.

Adapted from:
The Writing Center, University of North Carolina, Chapel Hill, *Writing in the Sciences* https://writingcenter.unc.edu/tips-and-tools/sciences/
BioMedical Editor, *Clear Science Writing: Active Voice or Passive Voice?* http://www.biomedicaleditor.com/active-voice.html

Advice for Building Competency in Scientific Writing

LEARN THE GENRES OF SCHOLARLY AND SCIENTIFIC WRITING

Throughout your career, you will engage with many genres (types) of writing. Scholarly and scientific genres are diverse and continually evolving. New forms emerge regularly (particularly in our digital and AI textual world). To develop genre skills, seek out excellent examples or models and study the structure of each genre—not just the content. Pay attention to arrangement, flow, word choice, and authorial (personal) style.

Academic Genres
Course papers, syllabi, presentations, program or curricular proposals, and more.

> **Emerging examples**: reflective learning journals, multimedia assignments, AI-assisted writing.

Scholarly Genres
Posters, journal articles, book chapters, encyclopedia entries, conference proposals, images/figures, abstracts, and more.

> **Emerging examples**: data papers, preprints, registered reports, graphical abstracts.

Professional Genres
Emails, cover letters, letters of recommendation, bio-statements, grant proposals, award applications, web pages, journal article peer reviews, and more.

> **Emerging examples**: LinkedIn profiles, research impact statements, digital portfolios, lab websites, AI-generated summaries.

Job Market Genres
Cover letters, recommendations, research statements, teaching philosophies, CVs, portfolios, and more.

> **Emerging examples**: video introductions, online teaching demos, AI-enhanced CVs, digital teaching portfolios.

CONSIDER WHEN YOUR WORD CHOICE is FIELD-SPECIFIC or JARGON

Be specific and avoid jargon. A reminder from: Helen Sword's *Stylish Academic Writing* (2012):

Academics turn to jargon for a wide variety of reasons: *to display their erudition, to signal membership in a disciplinary community, to demonstrate their mastery of complex concepts, to cut briskly into an ongoing scholarly conversation, to push knowledge in new directions, to challenge readers' thinking, to convey ideas and facts efficiently, and to play around with language. Many of these motivations align well with the ideals of stylish academic writing. Wherever jargon shows its shiny face, however, the demon of academic hubris inevitably lurks in the shadows nearby. Academics who are committed to using language effectively and ethically—as a tool for communication, not as an emblem of power—need first of all to acknowledge the seductive power of jargon to bamboozle, obfuscate, and impress.*

Recommendation: Read your draft with an eye to your motivation for using specialized language. Helen Sword suggests that *for every piece of jargon that you decide to keep, make sure you give your readers a secure handhold: a definition, some background information, a contextualizing word or phrase*. By the time you have clarified your usage, you might even find that you can let go of the word itself.

If you struggle with PASSIVE vs. ACTIVE VOICE

To start, here is a helpful refresher on sentence structure:

In **active voice** sentences, the subject *does* the action. (The subject is clearly the "agent")
The biologist bisected the specimen.

In **passive voice** sentences, the subject receives the action. (The agent is "elsewhere")
The specimen was bisected by the biologist.

In **passive voice** sentences, sometimes the agent is omitted.
The specimen was bisected. (The agent is absent and "presumed." Who is doing the bisecting?)

Arguments for ACTIVE Voice

- Active sentences are generally shorter and clearer.
- Active voice is more direct, engaged, and/or personal.
- Active voice "appropriately describes science."
- Many Journals prefer active voice.
- Passive voice sounds pompous & impersonal.
- Passive voice constructs ambiguous "agents."

Arguments for PASSIVE Voice

- Passive voice stresses what was done.
- Active voice requires personal pronouns.
- Passive voice is "more scientific." (crafting objectivity).
- Passive voice offers syntax (sentence structure) control. The writer can vary sentence structures for flow, connection, and interest.

CLARITY & CONCISION: Craft-to-expertise

Clear economical expression of research is the foundation of strong academic writing and the delight of every journal editor and faculty mentor. One of the most common complaints from journal editors and proposal reviewers alike about the quality of submissions is that the submissions are unclear, confusing, or verbose.

Writing with clarity and concision is a lifelong craft. And, while the challenge of writing with clarity and concision exceeds the advice that might fit in this box or our workshop, the following kind reminders can help:

- **Treat research writing as a craft**. You will continue to refine your science writing skills throughout your career.
- **Structure your research to "show**." A compelling narrative helps readers understand your work. Learn various ways scientific descriptions and findings can be arranged and pay attention to how they engage you as a reader. Remember, there's no single correct structure (but there are preferences, sometimes strong preferences).
- **Focus on sentence-level clarity.** Your research story is built from words and sentences. Use a style guide and study examples of edited writing.
    - Recommended options: Helen Sword's *The Writer's Diet and Stylish Academic Writing*
- **Avoid pompous or overly complex language**. Do not try to impress. Take care to revise drafts and remove academic egoism or verbal "virtuosity" before submitting.

FACULTY & PI MENTORS and GRAD & DOC MENTEES
Fostering a Writing Culture in Research Labs

While pursuing collaborative writing strategies, it is helpful to remember Peter Drucker's quip that "culture eats strategy for breakfast, lunch, and dinner." In other words, the best strategies can be undermined by obstructive collective processes and poor group dynamics. Ultimately, the most effective strategies for boosting collaborative writing skills and increase research outputs (manuscripts, proposal, and other scientific communications) are those that focus on lab culture.

Foster a culture through which lab/program members:

- Approach writing as a **process-oriented** activity, especially for developing graduate writers.
- Value and protect **dedicated time and space** for writing.
- Share writing at any stage, knowing it will be **thoughtfully and constructively assessed**.
- Recognize that every writer has **different capacities** for production and quality.
- View writing as a **necessary part of professionalization**—it may not always be enjoyable, but it should not be fearsome, daunting, or debilitating.
- Normalize seeking **assistance and feedback** as part of best practices in writing and mentoring.

(Adapted from Purdue Writing Lab, https://owl.purdue.edu/writinglab/faculty/documents/2021-Grad-Faculty-Guide.pdf)

Clarify writing plans (projects) & agreements (process) early on:

- Writing plans and agreements with graduate students need not be elaborate, but they should be **clear and documented** for easy reference. What is due, when, and to what quality standard?
- Keep plans **simple, clear, and flexible**.
- **Check in regularly** as agreed (weekly, biweekly, etc.)

Recognize the range of skills and backgrounds among your lab/program engage openly among lab/program members. Some options include:

1. Encourage the practice of **beginner's mind**. It is a smart way to interact, lead, and work together. Basically, you **listen actively and without judgement** to colleagues' experiences and ideas. This is easier said than done, and it takes practice, particularly when deadlines and pressures loom. Listening non-judgmentally allows you to accept what your mentor/mentee say about their experiences and for them to accept what you say about yours; in this way it is a powerful method of regular validation for team building. Listening non-judgmentally also allows you to **understand their ideas before jumping to conclusions** or automatically critiquing. Listening non-judgmentally **does not mean you always agree, like, or endorse** what your mentor/mentee has said, but because you listened openly and attentively, you will have fewer misunderstandings, and you are more likely to communicate well when disagreements arise.

2. Craft a **community agreement** to signal appropriate community behaviors and collegial support for writers. Review and revise each semester or when onboarding new team members. Here is a sample we have used in CSU Writes since 2020:
    - Be present, honest, authentic
    - Listen actively and with respect
    - Share speaking time (avoid dominating)
    - Encourage others as participants
    - Be open to and considerate of other perspectives
    - If uncertain, ask clarifying questions
    - If challenged, respond with grace
    - After our time together, share only what is yours to share (limit gossip)

Writing with Multilingual and Neurodivergent Coauthors

Collaborating with Multilingual Coauthors

Native English-speaking and multilingual (or English Language Learning) writer collaborations are common and highly valuable in research. The advice below can help you develop a mutually supportive and productive writing relationship with your coauthors whose first language is different than yours:

- **Normalize writing-as-learning.** None of us are born knowing academic English, and it is helpful to remember that all researchers will skill-build across their careers (no matter their first language).
- **Schedule time for iterative drafting.** Writing in multilingual collaborations often requires additional revision cycles. Plan accordingly.
- **Make document contents and expectations visible.** Clarify the purpose, audience, and structure of each section; share model papers or proposals for study and analysis of the genres and styles.
- **Give specific, actionable feedback on drafts.** Replace vague comments like "awkward" with precise explanations of what needs revision.
- **Prioritize content feedback before language issues.** Focus early-stage feedback on a document's macro level (purpose/argument, data, logic, value). Then address micro level issues during later revisions (grammar and style). *Note: an exception to "macro first" is if word-choice, grammar, or style issues inhibit the reader's understanding of the macro-level content. If purpose, logic, idea development are affected by micro issues, address those and provide explanation for revision.*
- **Avoid idioms and vague critique.** Expressions like "tighten this," "create flow," or "it needs punch" are unclear and do not address the specific textual issue. Use direct language about the requested revision instead.
- **Build language bridges**. Be curious about words and expressions that are meaningful to your multilingual colleague. Your linguistic world (and writing relationship) will expand meaningfully as well.

Collaborating with Neurodivergent Coauthors

You or your coauthor (or both of you) may be neurodivergent. If you followed the advice offered earlier in this handbook, you have already discussed what would best support you by reflecting on the questions in the guided **reflection conversation** (pp. 4-5) and you will soon **develop a Collaborative Writing Agreement** (pp.12-13). While one or both of you may not have feel comfortable disclosing a diagnosis, you and your coauthor should still clarify what you will need to contribute to a productive co-writing project and mentoring relationship. Neurodivergent researchers with ADHD, autism, dyslexia, or other cognitive differences benefit from transparent and predictable project structures and direct, clear communication.

These additional practices support inclusion without requiring personal disclosure, will benefit all researchers, and can be essential to a neurodivergent coauthor's success:

- **Offer a choice in communication** (e.g. shared documents, asynchronous comments, voice summaries)
- **Co-design work plans** that account for each other's specific needs
- **Draft timelines** that allow for work to be produced in manageable stages
- **Clarify who does what whe**n and make task tracking visible
- **Summarize and prioritize feedback** to reduce cognitive overload and prevent miscommunication.

Neuroinclusive collaboration benefits all writers by reducing ambiguity, improving workflow clarity, and creating a respectful writing environment grounded in mutual understanding and accountability (See Clancy et al., 2023 and Kendall-Taylor, 2025 in "Recommended Reads").

FACULTY & PI MENTORS and GRAD & DOC MENTEES

Developing Collaborative Writing Agreements

In addition to a lab handbook or code of conduct, developing a writing focused agreement can offer necessary support for you as coauthors. A collaborative agreement provides a shared understanding of your manuscript vision, writing project planning, and mentor/mentee writing relationship development.

Your responses to the following questions can assist you in crafting a mutually agreed upon set of writing-related objectives and guidelines to support your mentoring relationship. If you already have a mentoring agreement, select topics (for instance, when and how often to meet) may already be covered by your prior conversation and agreement. Even so, you may find it helpful set separate meetings dedicated to the writing project itself.

Feel free to adjust and add to the questions to suit your conversation and collaborative writing agreements:

1. **How often will we meet to discuss the writing project?**

   (***Some options***: *weekly, bimonthly, monthly, as needed*)

2. **How long should we plan to meet?**

   (***Some options***: *10-15 minutes, 30 minutes, 60 minutes, 2 hours, as long it takes*)

3. **Who is responsible for setting the meeting?**

4. **How far in advance should writing be submitted to receive advisor feedback?**

   (***Some options***: *24 hours, 2 days, 1 week, 2 weeks, more*)

5. **How often should writing be submitted to the advisor for feedback? And In what state should writing be submitted?**

   (***Some options***: *weekly loose drafts; monthly polished sections; multiple months polished manuscript/proposal, depends on the project and context*)

6. **What is the preferred method for submitting writing?**

   *(**Some options**: Email, Googledoc, Dropbox, OneDrive)*

7. **When should revised documents (based on feedback) be returned?**

   (***Some options***: *24 hours, 2 days, 1 week, 2 weeks, more*)

8. Grad Student Mentee: **What conversations or support would most support you as a writer?** (**consider** your writing practice, processes, products, projects)

9. Faculty Mentor: **What conversations or support would best support you as a writer?** (**consider** your writing practice, processes, products, projects)

10. **How might we create a meeting space of trust, authenticity, and clear communication?**

    (***Some options***: *be present; listen actively; keep feedback directed at task and document quality [not personal]; be open to multiple perspectives--particularly those that arise from differences across race, national origin, ethnicity, sex/gender identity, orientations, rank, appointment; if uncertain, ask clarifying questions; if challenged, respond with grace*.)

11. **What shall be kept confidential?**

    *(**Some options**: all information disclosed within the mentoring relationship and personal disclosures about writing challenges; Exceptions include "legal exceptions" that might require information about a participant to be disclosed to a third party in situations where a participant is believed to be a danger to self or others, where a participant is in need of immediate medical attention, or where a court order or subpoena requires disclosure.)*

12. **What will communicate our commitment to this writing collaboration of faculty advisor and graduate student?**

    *(**Some options**: print and sign 2 copies your responses to the questions comprising this "Mentor/Mentee Writer's Agreement." Add a statement of commitment: "I understand the effectiveness of this writing relationship is dependent upon my commitment to doing my part in drafting, revising, providing feedback, and in meeting regularly about our writing project[s]. I commit myself to doing so, barring illness or emergency.")*

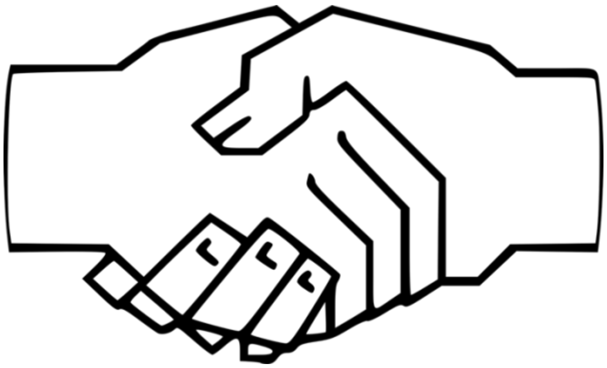

OpenClipart-Vectors, Pixabay

FACULTY & PI MENTORS and **GRAD & DOC MENTEES**
Coauthor Agreements

When your writing collaborations move from course- or lab-based projects into professional research documents that assign credit by author order (such as manuscripts, grant proposals, or technical reports), take time early on to talk openly about authorship and create a coauthor agreement for each project. Clarifying expectations for author order based on actual contributions makes collaboration more transparent, fair, and productive. These conversations may feel a bit awkward at first, but using a simple agreement (like the APA sample below) helps build trust, mutual accountability, and a healthy writing partnership. **Note**: the larger the author team, the more important these conversations will be. https://www.apa.org/science/leadership/students/authorship-agreement.pdf

Contract Regarding Publication Intent

We hereby enter into an agreement, as outlined below, regarding the publication of the project *tentatively* titled: ____

FIRST AUTHOR

Name (print): ____________________ Signature: ____________________

Percent effort: ________ Activity Score: ______

Brief description of basic responsibilities/role on project:

SECOND AUTHOR

Name (print): ____________________ Signature: ____________________

Percent effort: ________ Activity Score: ______

Brief description of basic responsibilities/role on project:

THIRD AUTHOR

Name (print): ____________________ Signature: ____________________

Percent effort: ________ Activity Score: ______

Brief description of basic responsibilities/role on project:

FOURTH AUTHOR

Name (print): ____________________ Signature: ____________________

Percent effort: ________ Activity Score: ______

Brief description of basic responsibilities/role on project:

It is agreed that authorship order may be renegotiated should an individual's responsibilities substantially change, or should an individual fail to perform their role as stated above. Furthermore it is agreed that if the project involves a student milestone, the manuscript (MS) or poster must be submitted for possible publication no later than 12 months from the date of the successful defense of the project. Should the manuscript not be submitted within 12 months time, it is agreed that the faculty supervisor will take primary responsibility for submission of the manuscript and will become first author.

Date contract signed: ________

Expected date of data completion: ________ Date project actually complete: ________

Expected date of MS/poster submission: ________ Date MS/poster submitted: ________

https://www.apa.org/science/leadership/students/authorship-agreement.pdf

FACULTY & PI MENTORS and GRAD & DOC MENTEES
Team Guidelines

If you are writing as part of a research team or on a long-term project where contributors may join, roles may shift, or students may graduate before publication, consider using a flexible authorship agreement that allows you to track contributions over time.

The sample below from Oliver et al. provides a manuscript management strategy for teams to map roles, responsibilities, and workflows in a transparent and accountable manner. Such a framework allows you to document evolving contributions, come to consensus on authorship order, and can help prevent misunderstandings as the project develops. Designed for multi-author research, it can be adapted to manage conflict, build momentum, and align writing productivity with team values.

Manuscript title: Insert title
Co-author list: Insert names
Target journal (tentative): Insert journal

Manuscript type: Select from these options, or add other: *(A) Disciplinary research article, (B) Multidisciplinary article, (C) Essay or commentary, (D) Data/Database paper, (E) Graduate-student led article, (F) Other (please specify)*

MS management strategy: To the degree that you know, select from these options or add other: *(A) Lone Wolf; (B) Dynamic Duo; (C) Board of Directors; (D) Round Table; (E) Organized Chaos; (F) Other (please specify)*

This document is intended to foster an open dialog on authorship that starts at the very beginning phase of a manuscript and carries through until manuscript submission and acceptance. We ask that all co-authors describe their contributions in the table below as a way to clearly define each co-author's responsibilities and accomplishments throughout the effort. We ask that in the early phases, you consider what components of the research effort you would like to contribute to; then, in the middle of the effort, to revisit your contributions; and finally, at the time of manuscript submission, we ask all co-authors to assess the contributions that they did. Using this information, the author-contribution statement will be written and reviewed by all authors.

Instructions: Please add your initials in the cell next to the contribution. Please also add a short-description of the activity. As a starting point, we recommend that co-authors participate in at least a single activity in 2 of the 4 major categories in the following table AND participate in a total of 3 activities combined; although, we expect there to be exceptions as well, some of which are identified below.

Activities	Author contributions
**Category 1: CONCEPT AND DESIGN**	
a) **Conceived of the MS idea/concept**– individually or collectively, helped to frame the overall idea for the MS, research questions, or scope; drafted conceptual figures or tables	
b) **Designed/outlined the MS** – individually or collectively helped to determine structure and content of the MS	
c) **Supervised co-authors and MS progress** – oversaw the MS progress	
d) **Other** -	

Access the full model with detailed instructions and rationale in *Appendix S1* of Oliver et al. *Ecosphere* (2018) "Strategies for effective collaborative manuscript development in interdisciplinary science teams."

GRAD & DOC MENTEES

FEEDBACK Stages and Types

Effective writing requires feedback, and different kinds of feedback are useful at different stages of the writing process. Below are strategies for seeking and using feedback as your draft evolves.

> **Quick Tip**: When sharing your draft, include a brief note about the stage of writing and the type of feedback you're seeking (see sample cover letter on page 18).

EARLY STAGES: General Reader-based Feedback (Non-expert)

Research writers can benefit from the feedback from a broad community of non-expert readers who may, or may not, have field-based knowledge. Have thoughtful non-experts or outside-of-discipline readers review short pieces (1-5 pages) for a draft's readability and clarity:

- Does the draft maintain the reader's interest?
- Is the point, purpose, or argument clear?
- Do ideas flow clearly from one sentence to the next?
- Where do reader's get stuck? Where does the draft need more detail or information?

MID STAGES: Criteria-based Feedback (Non-expert & Expert)

Writers can also rely on a network of expert or becoming-expert readers who can provide more in-depth feedback, perhaps on longer pieces of writing, about the MACRO structure (Big Picture) of the draft or about *micro* structure (sentence-level) quality of the writing. These can be peer colleagues, campus writing groups, committee members and advisors

To ask for MACRO or *micro* feedback, remember to tell reviewers (including mentors, advisors, and PIs) the type of feedback that would be most helpful for moving the manuscript forward:

MACRO issues:

- organization
- clarity
- gaps in logic or content
- redundancies

***micro* issues**:

- grammar
- punctuation
- style and tone

LATE STAGES: Expert Feedback (Field-Specific)

In final stages, ask expert readers (mentor, committee members, field experts) for field-specific insights, guidance, and corrections:

- scope and relevance of content
- accuracy of data, evidence, analysis
- strength of argument & development
- contribution to the field (including connections to existing literature)

FACULTY & PI MENTORS and GRAD & DOC MENTEES

Understanding Types of Feedback

Feedback can take many forms. Knowing what kind you are giving, receiving, or asking for can help you provide feedback and use it more effectively. Below are common types of instructional feedback with corresponding guidance on how to respond as a writer:

CORRECTIVE FEEDBACK

Reviewer		Writer	
**Modest to active effort**	Directly makes corrections on the page (e.g., grammar, punctuation, word choice), and may also revise sentence structure, idea development, concepts, tone, or style.	**Minimal effort**	Reviews and accepts changes but may not engage deeply with the reasoning behind them.

DIRECTIVE FEEDBACK

Reviewer		Writer	
**Active effort**	Identifies specific problems and offers suggestions for improvement but does not make the changes directly.	**Active effort**	Interprets suggestions and applies them to revise the draft.

INTERACTIVE FEEDBACK

Reviewer		Writer	
**Active effort**	Engages in dialogue with the writer by asking questions, offering commentary, and discussing areas of confusion or stylistic preference.	**Highly engaged**	Reflects on the feedback, makes decisions about how to address concerns and revises accordingly.

EVALUATIVE FEEDBACK

Reviewer		Writer	
**Modest effort**	Directly makes corrections on the page (e.g., grammar, punctuation, word choice), and may also revise sentence structure, idea development, concepts, tone, or style.	**Reflective to highly engaged effort**	Considers the feedback, decides what to revise, and may seek clarification or additional input.

(adapted from Purdue's *Faculty Guide: Working with Graduate Student Writers*
https://owl.purdue.edu/writinglab/faculty/documents/2021-Grad-Faculty-Guide.pdf)

GRAD & DOC MENTEES

Requesting Feedback: Cover Letter Method

When submitting a draft for feedback, you can facilitate a smoother and more productive review process from your (peer colleague, advisor/PI, committee member, or field expert) by including **a brief cover letter** with your document. This short note (typically 3–5 sentences or a concise paragraph) serves as a roadmap for the reader and sets expectations for the kind of feedback the writer is seeking.

Whether crafted as a formal request (a letter/email) or as an informal note on the first page of the draft, the "cover letter" should include these three elements:

1. **A brief summary of the draft**
   What is the document about?
   What is its purpose?
   Who is intended audience?
2. **A reflection on what's working well**
   What parts of the draft feel strong or complete?
   Where do you feel confident about the content or style/approach?
3. **A clear request for help**
   What specific areas are you struggling with or unsure about?
   What kind of feedback would be most helpful?
      macro (structure, clarity, argument)
      micro (grammar, style, tone)

Bonus: If appropriate, clarify the **feedback timeline** and when you would like to meet to discuss or if you need the review feedback by a specific date to meet a deadline.

Bonus-Bonus: Express **gratitude** and make sure your reviewer knows that you value their feedback and are that you are grateful for them making time to review your work.

Formal cover letter feedback template

Dear ________,

Thank you in advance for reviewing the attached draft of the third chapter of my dissertation on [topic, approach, purpose]. I plan to submit to [insert journal name] for a special issue by [insert date].

I think the introduction and literature review are fairly strong, and I have tried to clarify the value of [specific ideas] throughout.

I am still unsure about the organization of the discussion section and whether the connections I am making about [insert specific areas] are clear. Your insights about my descriptions of [insert specific areas from discussion section] as well as the section's overall structure and flow would be most helpful.

[your salutations]

Informal feedback request sample/template

Thank you for providing feedback on this draft article/dissertation chapter focused on [insert topic, approach, purpose]. The intro through methods is fairly strong (although, still in-process). It would be most helpful if you could focus on pages [insert #s]. The discussion feels disjointed, and I am concerned about readability. Talk with you on [date of meeting].

FACULTY & PI MENTORS
Communicating about Writing Feedback

When a graduate student or postdoc submits a draft with a cover letter, they are inviting you into a collaborative revision process. Your response can help guide their next steps and build their confidence as a writer. A thoughtful reply does not need to be long—it just needs to be clear, focused, and supportive.

What to include in a Response Cover Letter

1. **Gratitude and affirmation**
   Briefly affirm you read their note and understand what feedback would be most important.

2. **Summary of your feedback**
   Let the writer know how you addressed their concerns (corrections, marginal comments, general advice, etc.). If you provided additional feedback than requested, briefly describe the additional parts of the draft you focused on and why. This helps the writer understand your perspective and how to prioritize revisions.

3. **Top recommendations for revision**
   Offer 2–4 clear suggestions for improvement. These can be macro (structure, clarity, argument) or micro (grammar, style, tone), depending on what the writer requested.

4. **Optional: Offer to follow up**
   If appropriate, suggest a meeting or follow-up conversation to clarify feedback or discuss next steps.

Reviewer Response Example

Thank you for sharing your draft and cover letter. I focused on the discussion section as you requested and agree that your introduction and literature review are strong.

Here are my top recommendations:

1. *Strengthen transitions between major points in the discussion.*
2. *Clarify your argument in the final paragraphs.*
3. *Reduce redundancy across sections.*

Let me know if you'd like to meet to talk through these suggestions. I'm happy to help further.

Tips for Reviewers

Be specific: Vague comments like "this needs work" or "confusing" point to a place for revision, but they provide little guidance for how a writer might undertake or consider options for revisions. Identify what elements, sections, sentences, or ideas are troublesome and briefly explain why or describe what would work better.

Be constructive: Frame feedback as an opportunity for growth, not a critique of the writer or their ability.

Be mindful of tone: Even brief comments from a reviewer (particularly a supervisor or mentor) can have a big impact on a writer. Aim for clarity and encouragement. Remember, you both have goals of high-quality manuscript production and positive professional development through writing in common.

GRAD & DOC MENTEES
Writing Logs

Studies of writing productivity often recommend that writers keeping a log or writing journal to help them plan the steps of writing project, describe tasks for a writing session, identify a project's next steps, and, in some cases, to process ideas and feelings about the project. Robert Boice, researcher of procrastination and writer's block, identified **logs as an essential tool** for academics to regroup and build momentum on their writing projects (*Professors as Writers,* 1990).

Just as ship captains have used logbooks since ancient times to record the daily events of travel on the ocean—navigation, weather, crew management—writers use logs to record the status of their journey to craft the documents of research over an extended period of time (weeks, months, semesters, years).

Create a chart, open a spreadsheet, or use a notebook to track the work you complete in a session. Your writing log should reflect the overall status of your writing project as well as your writing practice.

In addition to logging dates of writing sessions and tasks completed, you may find tracking other elements of project momentum and professional development beneficial:

- writing targets (daily, weekly, monthly, project)
- start and stop times
- total time writing in a session
- accomplishments in a session (could be minor or major—all efforts count)
- number of words generated
- sections generated/edited
- emotions (feelings about project, wellbeing efforts)
- thoughts (insights, plans, directions)
- habits (writing routine, rituals)
- next steps
- to-dos before next writing session
- where to start at the next writing session (ideas, sections, etc.)

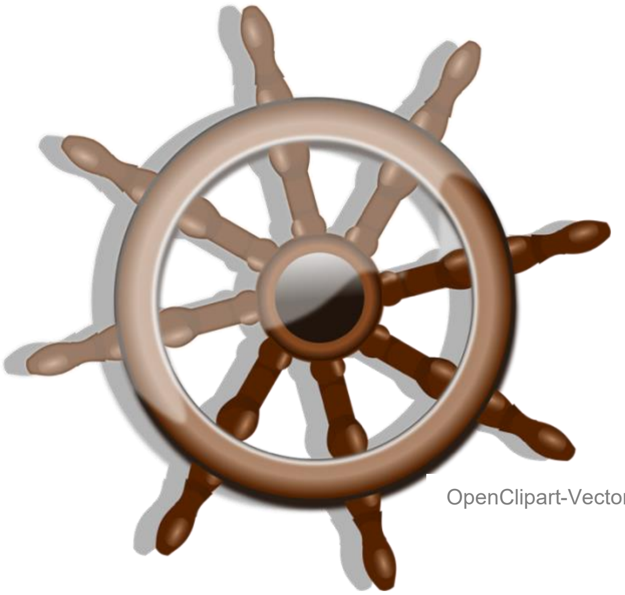

OpenClipart-Vectors, Pixabay

GRAD & DOC MENTEES
Error Trackers

Tracking sentence-level feedback can help you rapidly build field-specific or English vocabulary and improve the clarity of and overall readability of your research writing. A tracking process helps you target learning goals and monitor your progress. The chart and corresponding tracker bellow provide a sample method for categorizing and tracking errors from 10 manuscripts. It is designed to help you reflect on recurring issues, making revision processes and your professional development through writing intentional and more efficient.

Many of the “errors” listed use abbreviations and terms common in professional copyediting. Definitions have been provided.

Errors that **will interfere** with reader understanding	
unclear	The meaning is not clear. The sentence should be rewritten completely.
fragment	The sentence (thought) is incomplete.
run-on<br>comma splice	*Run-on*: two sentences joined without punctuation or transition<br>*Comma splice*: two complete sentences separated by a comma
verb tense	The verb is in the wrong tense. (problem with tense/mood/aspect)
syntax	Word order is incorrect or awkward.
wdch	The word does not say what the writer means it to say or convey. If significant, incorrect word choice (*wdch*) can affect the accuracy, precision, and general readability writing.
**Errors that are likely to interfere with reader understanding**	
transition<br>connection	The connector is incorrect or missing.
dangling or misplaced modifier	Descriptor (modifier) is too far from the word it modifies (i.e., is misplaced); the intended subject that is to be modified is absent (dangling modifier).
passive	The passive voice has not been arranged or used correctly.
pro/ant	Pronoun reference is not clear, or the pronoun doesn't agree with its antecedent (the noun to which it refers).
wdch	The word does not say what the writer means—to the degree it can impact the reader’s understanding.
// or parallel	Mixed or non-parallel structures of lists, comparisons.
**Errors less likely to interfere with reader understanding but affect the perceived quality of writing**	
AWK/non-idiom	The wording is non-idiomatic, sounds awkward or is difficult to understand.
prep	The wrong preposition is used, or the preposition is missing (e.g., *on, in, out, about, over, under, before, beyond, for, so,* and more).
sub/vb	The subject and verb do not agree.
**Least serious issues that can be addressed by careful editing**	
sp	spelling
punct	Punctuation is incorrect or missing.
art	Articles (*a, an, the*) are incorrect or missing.

**Manuscript**	1	2	3	4	5	6	7	8	9	10	Totals
**Errors that will interfere with reader understanding**											
unclear											
frag											
run-on											
bb tense											
syntax											
**Errors that are likely to interfere with reader understanding**											
tran/conn											
Dangling or misplaced modifier											
passive											
pro/ant											
wdch											
// parallel											
**Errors that are less likely to interfere with reader understanding, but affect the perceived quality of your writing**											
AWK/non-idiom											
prep											
sub/vb											
(other)											
**Less serious issues that should be addressed by careful editing**											
sp											
punct											
art											
**Totals**											
Total per/submission:											

(*developed by Dr. Cory Holland, 2016, and used in CSU Writes workshops and thesis writing courses*)

RESOURCES

The resources curated here are not intended to be comprehensive but, rather, to provide you with a quality start to your professional development through writing science with your mentor/mentee. The topics span authorship, mentoring and pedagogy, science writing, collaborative practice, and emerging guidance on GenAI. Many were featured in the CSU *Mentor through Writing* Workshop, and all offer practical support for faculty mentors and their graduate student or postdoctoral mentees. We invite you to explore what resonates with you and supports your current projects and writing partnerships. Start with the topics you find most interesting. Those resources are the most likely to serve you where you are as writers right now.

As your collaboration evolves and your scholarly practice develops, we encourage you to continue adding to this list by curating resources that reflect your shared values and research goals. Writing is an ever-changing process, and so are the relationships that sustain it. The rise of large language models (LLMs), for example, reminds us that we must continually adapt how we write, mentor, and collaborate.

Writing shapes our disciplines and extends the conversations of our fields. May the materials below support you in co-writing meaningful scholarship and making impactful contributions to research.

Books on Productivity, Science Writing, Graduate Student Skill Development

*Books cited in American Psychological Association (APA) format.

Allen, Jan. (2019) ***The Productive Graduate Student Writer***. Stylus Publications.

Cayley, Rachael. (2023) ***Thriving as a Graduate Writer: Principles, Strategies, and Habits for Effective Academic Writing***. University of Michigan Press.

Cayley, R., Coll, F., & Newman, D. A. (2025). ***Writing Together: Building Social Writing Opportunities for Graduate Students.*** University of Michigan Press.

Casanave, Christine Pearson. (2016) "What Advisors Need to Know about the Invisible 'Real-Life' Struggles of Doctoral Dissertation Writers." ***Supporting Graduate Student Writers: Research, Curriculum, and Program Design****.* University of Michigan Press.

Glasman-Deal, Hillary. (2020) ***Science Research Writing: For Native and Non-native Speakers*** *of English.* 2nd ed. World Scientific Publishing.

Goodson, Patricia. (2023) ***Becoming an Academic Writer: 50 Exercises for Paced, Productive, and Powerful Writing****.* 3rd ed. Sage Publications.

Heard, Stephen. (2016) ***The Scientist's Guide to Writing: How to Write More Easily and Effectively throughout Your Scientific Career****.* Princeton University Press.

Jensen, Joli. (2017) ***Write No Matter What: Advice for Academic Writers***. University of Chicago Press.

Kamler, Barbara and Pat Thomson. (2006) ***Helping Doctoral Students Write: Pedagogies for Supervision***. Routledge.

Schimel, Joshua. (2012) ***Writing Science: How to Write Papers that Get Cited and Proposals that Get Funded****.* Oxford University Press.

Swales, John and Christine Feak. (2012) ***Academic Writing for Graduate Students****.* 3rd ed. University of Michigan Press.

Sword, Helen. (2017) ***Air & Light & Time & Space****.* Harvard University Press.

AUTHORSHIP

Authorship Agreement Supplement. Graduate School, University of North Carolina, Charlotte. https://gradlife.charlotte.edu/wp-content/uploads/sites/1099/2024/03/FINAL-Authorship-Agreement-Supplement.pdf

A Graduate Student's Guide to Determining Authorship Credit and Authorship Order. APA Science Student Council, University of Notre Dame. (n.d.) https://psychology.nd.edu/assets/198758/ContractWorksheet.pdf

Oliver, S. et al. (2018) "**Strategies for effective collaborative manuscript development in interdisciplinary science teams**." Ecosphere. https://doi.org/10.1002/ecs2.2206

MENTORING and COLLBORATION

ADHD and Graduate Writing. *Writing Center. University of North Carolina, Chapel Hill. https://writingcenter.unc.edu/tips-and-tools/adhd-and-graduate-writing/*

Bommarito, D. V. (2015). **Collaborative research writing as mentoring in a U.S. English doctoral program.** *Journal of Writing Research*, 8(2), p. 267-299. https://doi.org/10.17239/jowr-2016.08.02.04

Center for the Improvement of Mentored Experiences in Research (CIMER): https://cimerproject.org/

Clancy, K., et al. (2023). **Neurodivergent graduate student writing experiences in STEM.** *Frontiers in Education*, vol. 8. https://www.frontiersin.org/articles/10.3389/feduc.2023.1295268/full

Faculty Guide for Working with Graduate Student Writers. Purdue Writing Lab. (2021) https://owl.purdue.edu/writinglab/faculty/documents/2021-Grad-Faculty-Guide.pdf

Faculty Guide for Working with Multilingual Student Writers. Purdue Writing Lab. (2021) https://owl.purdue.edu/writinglab/faculty/documents/2021-Multilingual-Faculty-Guide.pdf

"Guide for working with Non-Native English Writers." University of Minnesota. http://writing.umn.edu/sws/assets/pdf/WorkingNonnativeSpeakers.pdf

Jackson, V. (2024, May 12). **How to support neurodivergent postgraduate researchers**. *Times Higher Education*. https://www.timeshighereducation.com/campus/how-support-neurodivergent-postgraduate-researchers

Kendall-Taylor, N., & Nature Careers Editorial. (2025). **How to be a brilliant ally to your neurodivergent lab mate.** *Nature Careers*. https://www.nature.com/articles/d41586-025-00125-z

National Institutes of Health. (2023). **Disability inclusion in the biomedical research workforce**. https://diversity.nih.gov/programs-partnerships/disability-inclusion

Scientific Communication Advances Research Excellence (SCOARE): "Mentor Resource" https://www.scoareresources.com/for-mentors

GenAI and RESEARCH WRITING

Bjelobaba, S., et al. (2025). **Maintaining research integrity in the age of GenAI: an analysis of ethical challenges and recommendations to researchers.** *International Journal for Educational Integrity.* **21**:18 https://doi.org/10.1007/s40979-025-00191-w

Crilly, A., et al. (2025) **Ten simple rules for navigating AI in science**. PLOS Computational Biology. https://doi.org/10.1371/journal.pcbi.1013259

CSU AI Literacy Guide. Colorado State University. Morgan Library. https://libguides.colostate.edu/ai

Guidelines for Generative AI Use in Graduate Studies. University of Calgary. https://grad.ucalgary.ca/sites/default/files/teams/1/Guidelines%20for%20Generative%20AI%20Use%20in%20Graduate%20Studies%20April%202025.pdf

International Association of Scientific, Technical & Medical Publishers (STM. (2023) **Generative AI in Scholarly Communications: Ethical and Practical Guidelines for the Use of Generative AI in the Publication Process.** https://s3.eu-west-2.amazonaws.com/stm.offloadmedia/wp-content/uploads/2024/08/10031822/STM-GENERATIVE-AI-PAPER-2023-1.pdf.

International Association of Scientific, Technical & Medical Publishers (STM). (2025) **Recommendations for a Classification of AI Use in Academic Manuscript Preparation.** https://s3.eu-west-2.amazonaws.com/stm.offloadmedia/wp-content/uploads/2025/04/23020709/STM_AI_Classification_Recs_19_Sept2025-1.pdf.

Research with Generative AI. Harvard University. https://www.harvard.edu/ai/research-resources/

Seckel, E., (2024) **Ten simple rules to leverage large language models for getting grants**. PLOS Computational Biology. https://doi.org/10.1371/journal.pcbi.1011863

STM Association. (2025) **Recommendations for a Classification of AI Use in Academic Manuscript Preparation**. https://s3.eu-west-2.amazonaws.com/stm.offloadmedia/wp-content/uploads/2025/04/23020709/STM_AI_Classification_Recs_19_Sept2025-1.pdf

Weaver, Kari. (2024) **The Artificial Intelligence Disclosure (AID) Framework.** College and Research Libraries News. https://crln.acrl.org/index.php/crlnews/article/view/26548/34482.
