# Supplementary material for: Ten simple rules for an effective mentor–mentee writing partnership
Source: PLoS Comput Biol. 2026 May 6;22(5):e1014250. doi: 10.1371/journal.pcbi.1014250 (PMC13148713; doi:10.1371/journal.pcbi.1014250)
Supplement: S2 File — A template for developing an agreement on allowed uses of AI during preparation of a manuscript or proposal. (PDF) [file pcbi.1014250.s002.pdf]

CSU WRITES
COLORADO STATE UNIVERSITY

Developed by Dr. Kristina Quynn, Director CSU Writes
Dr. Carol Wiluz, Director CSU CMB

Please share AI collaborative agreements widely to support AI transparency and ensure research integrity

AI-Use Agreement for Coauthors Template

Title of Manuscript: [Insert Title]
Coauthors: [List Names]
Date: [Insert Date]

1. Purpose of Agreement

This agreement establishes guidelines for the ethical and responsible use of Artificial Intelligence (AI) programs in the collaborative writing of [Insert Manuscript Title]. All coauthors agree to adhere to these terms to ensure academic integrity and transparency.

2. Approved AI Programs & Uses

Coauthors agree to use the following AI programs for the specified purposes:

Permitted Use of AI or other Platforms	Authorized platforms	Secure for unpublished data?*	Explanation/Qualification
Improvement of grammar, spelling, style, formatting	Grammarly<br>MS Word	Yes	Text must be generated first by a human, and edits must undergo human review.
Refining content and structure of a manuscript	MS CoPilot<br><br>Gemini 2.5	Only if logged in to CSU MS365<br><br>No	e.g. reverse outlining, identifying problems with consistency, voice, organization, etc..Making textual suggestions (i.e., editorial) but not contributing ideas (i.e., not authorial)
Generation, editing or correction of images for illustrative purposes	DALL-E	No	Only for images created as an illustration, not for data visualization or representation output.
Literature review	NotebookLM<br>Elicit	N/A	For identifying and summarizing sources. Serving as a knowledge bank for brainstorming ideas. All sources and information verified by a human. Not for final text generation.
Reference management	Zotero	Yes	For generating bibliography. Human review for formatting and accuracy.

*Sharing unpublished information with an LLM includes the possibility that it will be used in training new models. You may want to limit the use of some platforms to sections of the manuscript that do not contain novel or sensitive information (e.g., selections from the Introduction, Materials & Methods).

For all AI programs, coauthors must agree in advance and provide necessary processes for tracking plans for disclosure.

3. Ethical AI Use Guidelines

- **Transparency:** Any AI use must be tracked and disclosed according to journal or funder guidelines.
- **Human Oversight:** No AI-generated content will be included without human verification, revision, and approval.
- **AI-Generated Citations:** AI programs may fabricate or hallucinate references; all sources must be manually checked and confirmed.
- **No AI in Data Interpretation:** AI will not be used for interpreting results or drawing conclusions.
- **Data Security & Confidentiality:** No unpublished, confidential, sensitive, or regulated research data will be uploaded to an AI program; only institution-approved secure systems may be used when working with research data.
- **Institutional & Journal Compliance:** AI use must align with submission policies and university ethical guidelines.

4. AI Tracking and Disclosure

To ensure the ethical use of AI described above, we will maintain a record of all AI use and disclose it in accordance with journal, funder, and institutional requirements. For manuscript drafting and editing, we will document: the purpose of use, AI tool and model/version, date, prompt(s), and resulting output. For research-related AI use (e.g., study design, data collection, data processing/coding, modeling, or analysis), we will clearly describe methods in the manuscript body and provide appropriate citations and documentation to support transparency and reproducibility.

5. AI-Free Final Review (Human Review)

Before submission, all coauthors agree to conduct a final **AI-free or Human review** to:

- Ensure the manuscript reflects original scholarly contributions.
- Verify citations, references, and AI-assisted summaries.
- Maintain coherence and authorial voice.
- Confirm accuracy and integrity of data, results, and interpretations.
- Check for AI-generated errors, bias, or fabricated content.
- Ensure compliance with journal, funder, and institutional AI-use policies and disclosure requirements.

6. Dispute Resolution

If concerns arise regarding AI use in the manuscript, coauthors will:

1. Discuss concerns as a team and agree on revisions.
2. Seek guidance from an ethics officer or institutional writing support if necessary.
3. Ensure compliance with publishing and institutional guidelines.

7. Agreement & Signatures

By signing below, each coauthor agrees to abide by the ethical use of AI programs as outlined in this document.

Coauthor Name	Signature	Date
[Name]	[Signature]	[Date]
[Name]	[Signature]	[Date]
[Name]	[Signature]	[Date]

Appendix: AI and Research Resources

Bjelobaba, S., et al. (2025). **Maintaining research integrity in the age of GenAI: an analysis of ethical challenges and recommendations to researchers.** *International Journal for Educational Integrity.* **21**:18 https://doi.org/10.1007/s40979-025-00191-w

Crilly, A., et al. (2025) **Ten simple rules for navigating AI in science**. PLOS Computational Biology. https://doi.org/10.1371/journal.pcbi.1013259

Seckel, E., (2024) **Ten simple rules to leverage large language models for getting grants**. PLOS Computational Biology. https://doi.org/10.1371/journal.pcbi.1011863

STM Association. (2025) Recommendations for a Classification of AI Use in Academic Manuscript Preparation. https://s3.eu-west-2.amazonaws.com/stm.offloadmedia/wp-content/uploads/2025/04/23020709/STM_AI_Classification_Recs_19_Sept2025-1.pdf

Weaver, Kari. (2024) **The Artificial Intelligence Disclosure (AID) Framework.** College and Research Libraries News. https://crln.acrl.org/index.php/crlnews/article/view/26548/34482.

Artificial Intelligence Disclosure (AID) Statement
Artificial Intelligence: ChatGPT v.4o; *Conceptualization*: ChatGPT was used to identify agreement categories.
